# The complete mitogenome of *Caulerpa lentillifera* and its phylogenetic analysis

**DOI:** 10.1080/23802359.2019.1667906

**Published:** 2019-09-23

**Authors:** Xuli Jia, Tao Liu, Xumin Wang, Xianming Tang, Yuemei Jin

**Affiliations:** a Laboratory of Genetics and Breeding of Marine Organism, College of Marine Life Sciences, Ocean University of China, Qingdao, Shandong, People’s Republic of China;; b Hainan Academy of Ocean and Fisheries Sciences, Haikou, Hainan, People’s Republic of China;; c Hainan Provincial Key Laboratory of Technology for Tropical Seawater Aquaculture, Haikou, Hainan, People’s Republic of China;; d College of Life Sciences, Yantai University, Yantai, Shandong, People’s Republic of China

**Keywords:** *Caulerpa lentillifera*, mitogenome, phylogenetic analysis

## Abstract

*Caulerpa lentillifera* is a marine nutrient-rich edible green algae, with its external shape similar to ‘sea grape’, it has functions of purifying blood, anti-oxidation, anti-cancer, and anti-tumor. The mitogenome sequence of *C. lentillifera* is 209,894 bp long. A total of 67 genes were determined, including 17 protein-encoding genes, 3 rRNA genes, 27 tRNA genes, and 20 unidentified open reading frame (ORF). Phylogenetic analysis showed that *C. lentillifera* clustered together into a single branch. The mitogenome analysis will help the understanding of Ulvophyceae evolution.

*Caulerpa lentillifera* is a kind of nutrient-rich edible green algae and it contains a variety of amino acids and vitamins needed by the human body (Guo et al. 2015). It is also known as ‘green caviar’ in Japan and is widely distributed in the tropical waters of the tropical edible economic algae. *Caulerpa lentillifera* belongs to Chlorophyta, Ulvophyceae, Bryopsodales, Caulerpaceae, Caulerpa. It is also effective in treating hyperglycemia, anti-oxidation, and enhancing immunity (Maeda et al. 2012; Paul et al. 2014). In this study, we sequenced, assembled, and annotated the mitochondrial DNA of *C. lentillifera,* conducted a systematic genome study to evaluate the evolutionary trend of mitochondrial DNA, compared with others submitted in NCBI, and studied the phylogenetic relationship between *C. lentillifera* and other green algae.

The determination of the complete *C. lentillifera* mitogenome sequence by next-generation sequencing methods was conducted. The specimen was collected from north China (Qionghai, Hainan Province, 19°14′36.06″N,110°28′13.01″E) and stored at the Culture Collection of Seaweed at the Ocean University of China (accession number: 2018050001). Total DNA was extracted using the modified CTAB method (Doyle and Doyle 1990). Paired-end reads (150 bp) were sequenced by using Illumina HiSeq system (Illumina, San Diego, CA) and Pacbio system. tRNAscan-SE Search Server (Schattner et al. 2005) was used to identify the tRNA genes. The other mitogenomic regions were annotated with Geneious R10 (Biomatters Ltd, Auckland, New Zealand), using the *C. lentillifera* (NC_038217) mitogenome as reference.

The complete *C. lentillifera* mitogenome is a circular DNA molecule measuring 209,894 bp in length with the overall G + C content of 50.9% (GenBank accession number:MN201586). The mitogenome contains 67 genes, including 17 protein-coding, 3 rRNA, 27 tRNA genes, and 20 unidentified open reading frame (ORF). The nucleotide composition was 24.92% A, 25.81% C, 25.08% G, and 24.19% T. The length of the coding region was 106,258 bp, corresponding to 50.62% of the total length. Of the 17 protein-coding genes, 9 (52.94%) ended with the TAA stop codon, 4 (23.53%) with TAG (*cox*1, *cytb, ND2*, and *ND6*) and 4 (23.53%) with TGA(*atp6*, atp9, *cox3*, and ND4). All protein-coding genes in *C. lentillifera* were found to use the start codon ATG. The lengths of 3 rRNA genes are 2214 bp (*rrn23 rRNA*), 110 bp (*rrn5 rRNA*), and 1454 bp (*rrn16 rRNA*). The gene numbers and structures were largely similar to *C. lentillifera* published in the NCBI database, but it is 860 bp longer than the reference, it was verified by amplification experiment that the 860 bp was indeed present in the mitochondrial genome of *C. lentillifera*, which is correct.

Five shared mitochondrion protein sequences from 39 green algae and 1 outgroup including *C. lentillifera* were used to conduct phylogenetic analysis by using MrBayes 3.1.2 software (Ronquist and Huelsenbeck 2003). *Galdieria sulphuraria* strain 074W(NC_024666) served as the outgroup. Poorly aligned regions were removed by using the Gblocks server (Castresana 2000). *Caulerpa lentillifera* clustered together into a single branch and showed a further relationship with Ulvophyceae species (Figure 1). This complete mitogenome analysis of *C. lentillifera* helps us better understand the evolutionary process of Ulvophyceae.

**Figure 1.**
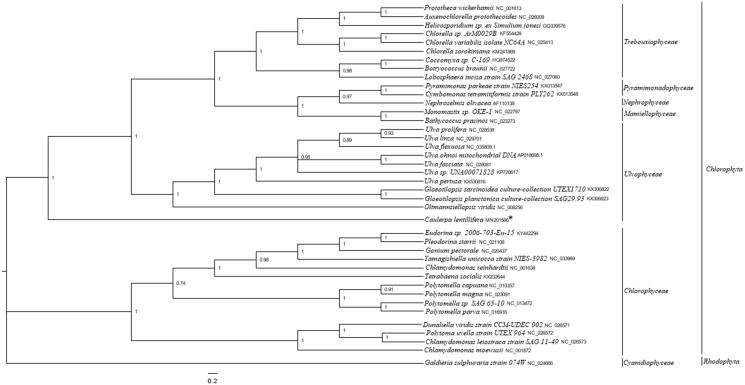
Phylogenetic tree (Bayesian inference) based on complete mitogenomes of Chlorophyta. Support values for each node were calculated from Bayesian posterior probability (BPP). Asterisks following species names indicate newly determined mitogenomes.
